# Optimizing plant spatial competition can change phytohormone content and promote tillering, thereby improving wheat yield

**DOI:** 10.3389/fpls.2023.1147711

**Published:** 2023-03-06

**Authors:** Pan Liu, Baozhong Yin, Xuejing Liu, Limin Gu, Jinkao Guo, Mingming Yang, Wenchao Zhen

**Affiliations:** ^1^ College of Agronomy, Hebei Agricultural University, Baoding, China; ^2^ State Key Laboratory of North China Crop Improvement and Regulation, Baoding, China; ^3^ Key Laboratory of North China Water-saving Agriculture, Ministry of Agriculture and Rural Affairs, Baoding, China; ^4^ Key Laboratory of Crop Growth Regulation of Hebei Province, Baoding, China; ^5^ College of Plant Protection, Hebei Agricultural University, Baoding, China; ^6^ College of Clinical Medicine, North China University of Technology, Tangshan, China; ^7^ Wheat Research Center, Shijiazhuang Academy of Agriculture and Forestry Sciences, Shijiazhuan, China; ^8^ College of Agronomy, Northwest A&F University, Xianyang, China

**Keywords:** winter wheat, line-spacing shrinkage and row-spacing expansion, phytohormone, tiller, grain yield

## Abstract

As an important type of interplant competition, line-spacing shrinkage and row-spacing expansion (LSRE) can increase the number of tillers and improve resource utilization efficiency in wheat. Wheat tillering is closely related to various phytohormones. However, it is unclear whether LSRE regulates phytohormones and their relationship to tillering and wheat yield. This study evaluated tillering characteristics, phytohormone content in tiller nodes at the pre-winter stage, and grain yield factors for the winter wheat variety Malan1. We used a two-factor randomized block trial design with two sowing spacings of 15 cm (15RS, conventional treatment) and 7.5 cm (7.5RS, LSRE treatment) at the same density and three sowing-date groups (SD1, SD2, and SD3). LSRE significantly promoted wheat tillering and biomass at the pre-winter stage (average increases of 14.5% and 20.9% in the three sowing-date groups, respectively) and shortened the accumulated temperature required for a single tiller. Changes in the levels of phytohormones, including decreased gibberellin and indole acetic acid and increased zeatin riboside and strigolactones, were determined by high-performance liquid chromatography and were shown to be responsible for the tillering process under LSRE treatment in winter wheat. LSRE treatment can improve crop yield by increasing the number of spikes per unit area and grain weight. Our results clarified the changes in tillering and phytohormones content of winter wheat under LSRE treatment and their correlation with grain yield. This study also provides insights into the physiological mechanisms of alleviating inter-plant competition to improve crop yield.

## 1 Introduction

Wheat (*Triticum aestivum* L.) is one of the three major food crops worldwide (Yang et al., 2018) and the primary food source for more than one-third of the world’s population. In particular, as the global population increases (expected to reach 10 billion by 2050), wheat demand will increase further (Cai et al., 2018). The North China Plain (NCP) is the largest wheat-producing area in China, accounting for approximately 56% of the total output, and plays a critical role in ensuring China’s food security (Feng et al., 2023). The main planting system adopted by the NCP is double cropping of winter wheat and summer maize each year. This can improve the land multiple cropping index and effectively increase grain yield. However, this method has also postponed the winter wheat sowing date in this area, which is affected by the previous maize harvest date, weather, soil moisture, and other factors, resulting in an insufficient crop population and reduced resistance during the pre-winter stage (Xia et al., 2023). This situation is even more prominent in the heat resource shortage area north of the NCP. The ability to use limited heat resources to generate sufficient populations and healthy individuals is important for understanding the theory and technology of high-yield wheat cultivation in this region.

Tillering is a crucial agronomic characteristic for controlling the population as well as the individual structure and yield of wheat and other cereal crops (Fioreze and Rodrigues, 2014; Lecarpentier et al., 2019). Tillers have essential functions in organic matter storage and should be present in an appropriate quantity and quality to guarantee the number of effective ears in wheat fields. Tillers also promote the development of secondary roots, improve the utilization efficiency of water and nutrients on farmland, and ultimately affect yield (Yu et al., 2020). Therefore, regulating the growth and development of tillers, particularly the quantity and quality of tillers in the pre-winter stage, is essential to ensure high-yield and high-efficiency wheat cultivation. Wheat tillers develop from axillary meristems, and genetic factors mainly control their establishment and axillary bud formation. Axillary meristem growth is a typical quantitative genetic trait regulated by a complex regulatory network involving genetics, hormones, and the environment (Wang et al., 2016; Barbier et al., 2019).

Tillering of gramineous crops is initiated from the base of the plant, and the growth and development of tillers are significantly affected by the distance and density of adjacent plants (Lecarpentier et al., 2019). Therefore, spatial competition resulting from the individual distribution of plants is a critical environmental condition affecting the growth of wheat tillers (Yu et al., 2020). High-density planting inhibits the formation of wheat tiller buds and reduces the number of tillers per plant (Xu et al., 2016; Barbier et al., 2019). In addition, even at the same planting density and row spacing, wheat tillers exhibit differences. For example, at an equal planting density, moderately reducing row spacing will promote tillering, although row spacing that is too narrow will prevent tillering (McSteen and Leyser, 2005; Ozturk et al., 2006). Because wheat plant spacing is much smaller than row spacing, competition between plants in the same row is more significant than that between rows for wheat tillering. Therefore, wheat tillering can be promoted appropriately by reducing row spacing, expanding plant spacing, and alleviating plant competition. These control measures have become more practical in fields with fewer tillers because of late sowing dates and low accumulated temperatures.

Various phytohormones participate in the regulation of branch and tiller development (Müller and Leyser, 2011; Wang et al., 2018). For example, indole-3-acetic acid (IAA) and cytokinin (CTK) respectively promote and inhibit rice lateral bud formation. Other studies have also shown that the growth of wheat tiller buds is regulated by the concentrations of IAA, ABA, and zeatin (ZT) in tiller nodes, and the ratio of the contents of these phytohormones also plays a significant role (Shang et al., 2021). Strigolactones (SLs) are carotenoid-derived phytohormones that were first demonstrated in 2008 to inhibit the outgrowth of axillary buds (Liu et al., 2011; Liu et al., 2021b). Although discovered later, the effects of SL hormones play a crucial role in regulating the tillering of gramineous crops. Thus, their functions in different varieties of wheat and whether they have antagonistic or promotional relationships with other phytohormones should be examined.

The degree of competition between phytohormones and individual plant spaces significantly affects the tillering of gramineous crops and ultimately affects crop yield (Lafond, 1994; McSteen and Leyser, 2005; Evers et al., 2006; Hussain et al., 2012). However, there is little research on how phytohormones respond to plant competition at different tillering stages and how they regulate tillering and yield formation (Müller and Leyser, 2011). To understand these pivotal factors, we conducted a two-year field experiment in the northern part of the NCP, where heat resources are relatively scarce. We aimed to systematically examine the effects of line-spacing shrinkage and row-spacing expansion (LSRE) treatment on tillers and phytohormones of winter wheat at different sowing dates under the same sowing density as the different tillering effects on grain yield factors. This study provides some new ideas for expanding tillering regulation and efficiently utilizing light and heat resources, particularly for winter wheat production in areas with limited heat resources.

## 2 Materials and methods

### 2.1 Overview of test site

**Figure 1:**
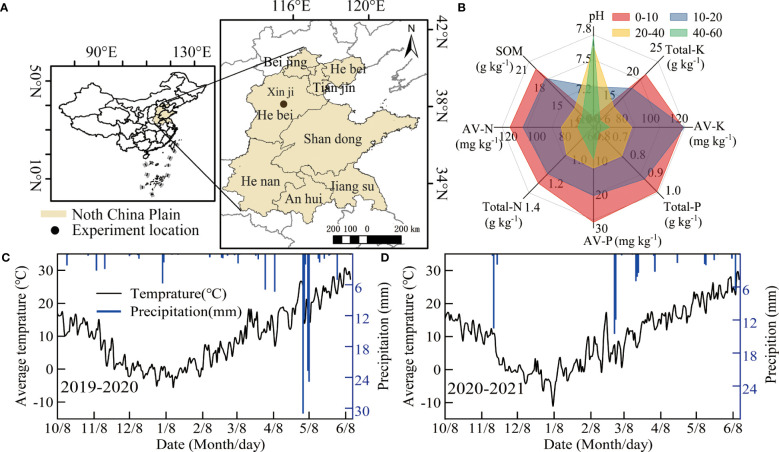
Basic information of the test site: **(A)** distribution of test sites and **(B)** foundation soil capacity of the test field. **(C, D)** are the daily precipitation and average temperature during the test.

A two-year field experiment was performed during the winter wheat growing season (from early October to mid-June of the following year from 2019 to 2021) at the Malan R&D base of the National Grain High Yield Science and Technology Project in the NCP (Xinji City, Hebei Province, China, 115.22°E, 37.92°N, 43 m above sea level). The location and foundation soil fertility of the test station are shown in **Figures 1A, B**. From 1991 to 2018 at the test station, the average annual precipitation and temperature were 458.9 mm and 12.9°C, respectively; these values during the winter wheat season were 122.0 mm and 12.5°C, respectively. The precipitation and daily average temperature from 2019 to 2021 are shown in **Figures 1C, D**. The cumulative precipitation in the two winter wheat seasons was 139.8 mm and 72.4 mm, respectively.

### 2.2 Experimental design and treatments

**Figure 2:**
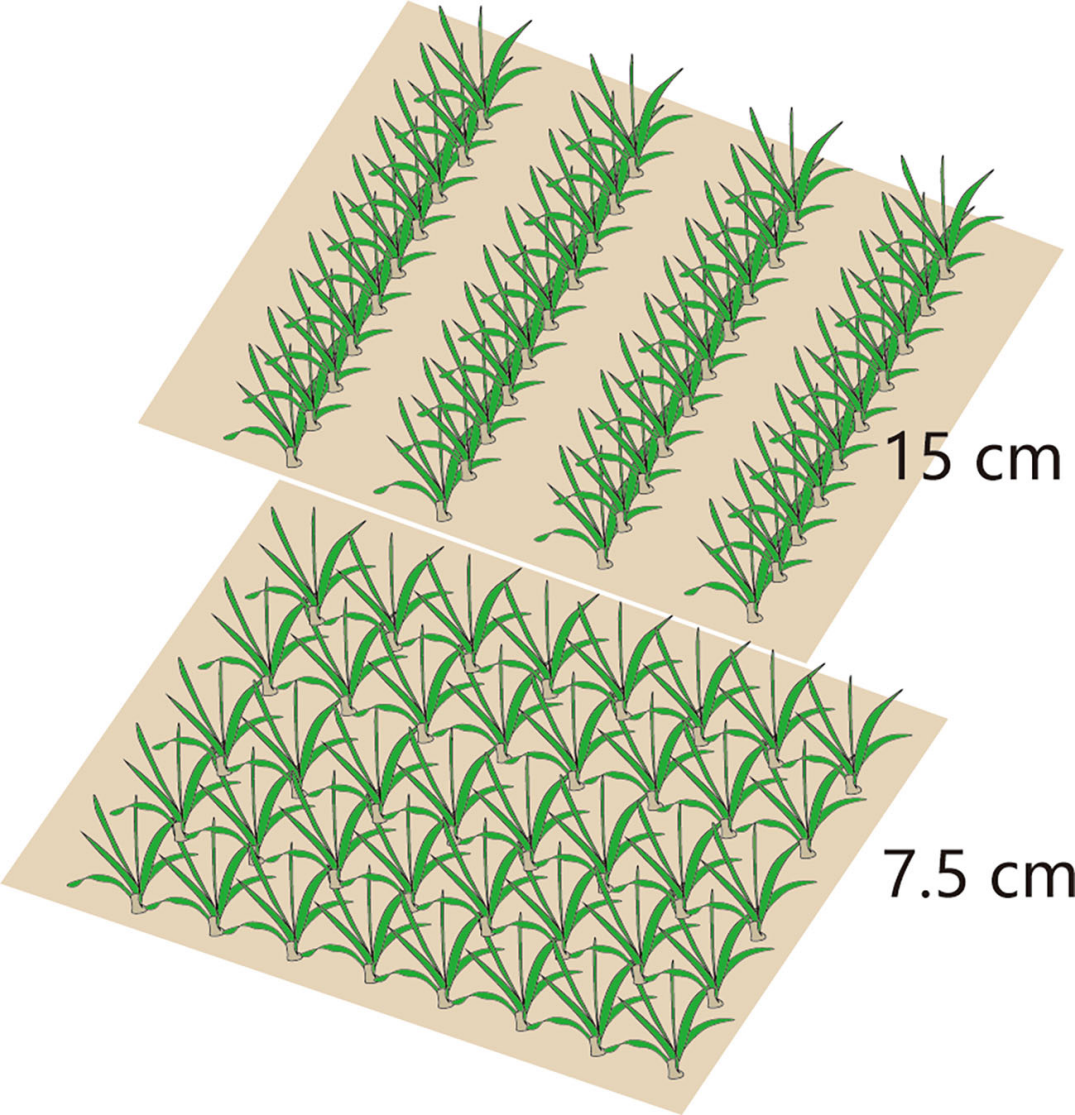
Spatial distribution of plants in different row spacing.

A two-factor randomized block design was adopted: factor I involved a row spacing of 15 cm (15RS, conventional row spacing for wheat seeding in the NCP) and 7.5 cm row spacing (7.5RS, LSRE treatment). The field plant distributions for the two types of row spacing are shown in **Figure 2**. Factor II included a suitable sowing date (8 October, SD1), delayed sowing date 1 (13 October, SD2), and delayed sowing date 2 (18 October, SD3). The area of each plot was 65 m^2^ (10 m × 6.5 m). The sowing densities of the two-row spacing treatments were the same on each sowing date. We selected Malan 1 (Variety approval No.: Jishenmai 20218011), a semi-compact winter wheat variety that is widely planted in the northern part of the NCP, as the test variety. The basic seedling design of three sowing-date groups (SD1, SD2, and SD3) was 3.3 × 10^6^ kg hm^−2^, 3.75 × 10^6^ hm^−2^, and 4.05 × 10^6^ hm^−2^. Their corresponding seeding amounts were 180.0 kg hm^−2^, 210.0 kg hm^−2^, and 225.0 kg hm^−2^, respectively. After harvesting the previous summer’s maize, the straw was crushed and returned to the field, and a 1GQN-230B rotary cultivator was used twice at a depth of 15 cm. Before sowing, 120 kg hm^−2^ of pure nitrogen, 112.5 kg hm^−2^ of P_2_O_5_, and 112.5 kg hm^−2^ of K_2_O were added. Pure nitrogen was applied per hectare during the first irrigation in the spring. A Trimer Pico 64 portable soil moisture meter (TDR, IMIKO, Bochum, Germany) was used to measure soil moisture content (V/V). The target water content of irrigation at different stages of the wheat field was 80% of the field capacity (V/V); the calculated soil depth of irrigation before sowing was 0–40 cm, and the 0–60 cm soil layer for the jointing and anthesis stages. The irrigation amounts at different growth stages are shown in **Table 1**.

**Table 1:** Irrigation amount of wheat fields under different treatments (m^3^ hm^−2^).

Row space (cm)	Sowing date	2019–2020		2020–2021		
		Before sowing	Jointing stage	Before sowing	Jointing stage	Anthesis stage
15	SD1	538.5	873.0	384.0	631.5	826.5
	SD2	552.0	852.0	412.5	606.0	831.0
	SD3	580.5	798.0	423.0	568.5	790.5
7.5	SD1	568.5	822.0	336.0	591.0	756.0
	SD2	594.0	766.5	388.5	564.0	733.5
	SD3	550.5	723.0	393.0	525.0	678.0

No irrigation in the anthesis stage of wheat in 2020.

### 2.3 Number of basic seedlings and tillers

**Figure 3:**
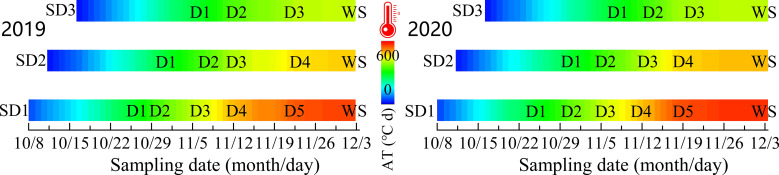
Sampling and determination date at pre-winter stage under different sowing dates.

For each repeated plot, three rows of wheat exhibiting uniform growth were randomly selected as the survey sample section at the trilobal stage (BBCH 13, Cornelius et al., 2011) for each treatment and the basic number of wheat seedlings per unit area (BSN). We investigated the tiller number for every 60–80°C d increase in the active accumulated temperature (daily average temperature ≥3.0°C, Equation 1) from the trilobal stage to the overwintering stage (WS), and the survey data were recorded as D1, D2, D3,… Dn, WS, respectively (the specific measurement date is shown in **Figure 3**). The method used to calculate the active accumulated temperature (AT) in the prewinter stage is shown in Equation 2. From the standing stage of the wheat, the investigation was conducted once every 2–3 days until the number of tillers reached its maximum (BBCH 29) in spring. To avoid the impact of the survey on wheat growth, we only investigated one sample section of one repeated plot each time and alternated the measurement of different sections: tillers formed in winter (TP) and spring (TS) at the survey sample points were marked with red and yellow nylon threads (Tilley et al., 2019), respectively. When the tiller number reached its maximum, the sum was determined as the total tillers (PT) for the entire growth period.

**Figure 4:**
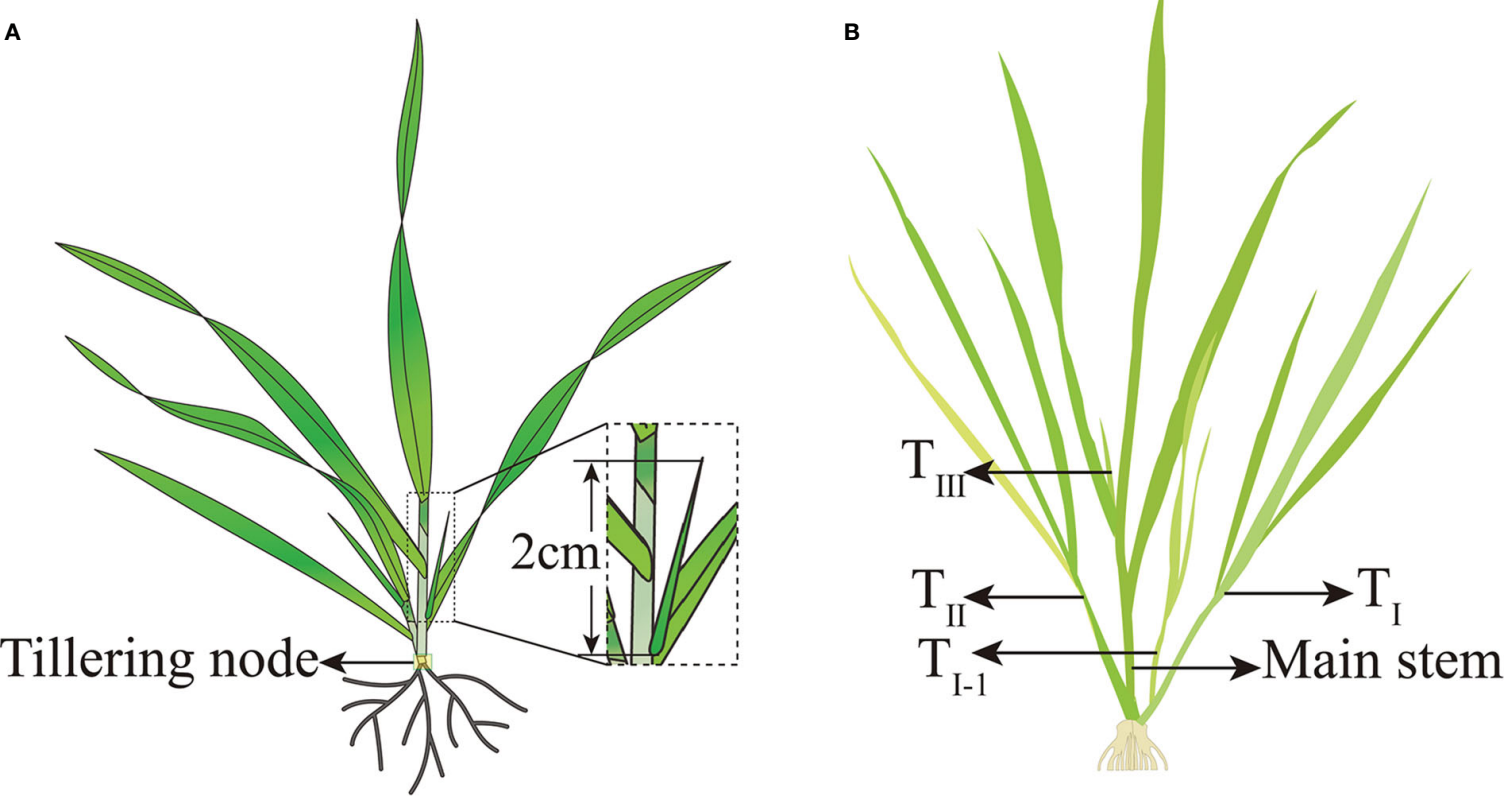
Sampling position, tiller criteria, and nomenclature of tillers for wheat. **(A)** Sampling position and tiller criteria for wheat; **(B)** nomenclature of tillers for wheat.


A tiller was considered to have formed when the tiller bud protruded 2 cm from the base leaf axil (**Figure 4A**), and the tillering date of the entire field was considered when 50% of the plants reached this standard. The AT required for tillering formation is given by Equation 2. According to the sequence of primary tillering of the main stem, all tillers were named T_I_, T_II,_…T_n_ and the secondary tillers were named T_I-1_, T_I-2,_ and so on (**Figure 4B**). The green stalk number per unit area (N_GS_) is the sum of the main stems and tillers (Equation 3).

Equation 1 $$AT\left(\text{°C }d\right)=\sum_{i=1}^{n}t_{i}(\text{when }t_{i}<3,\;t_{i}=0)$$

Equation 2 $$AT_{n}\left(\text{°C }d\right)=AT_{s\to n}-AT_{s\;\to \;\left(n\;-\;1\right)}(\text{n}\geq 1)$$

Equation 3 $$N_{GS}\left(stalks\;m^{-2}\right)=N_{MS}\left(main\;stems\;m^{-2}\;\right)+N_{T}\left(tillers\;m^{-2}\;\right)$$

where AT represents the active accumulated temperature from sowing to the *i*th day, n is the number of days after sowing (d), and *t_i_* is the daily average temperature (Â °C) on the *i*th day after sowing.

A*_n_* represents the AT required to form the *n_th_* main stem tiller, AT*_s → n_* and AT*_s → (n − 1)_* represent the AT required from sowing to the *n*th and (*n − 1*)th main stem tiller formation, respectively. When *n* = 1, A*_1_* is the AT required from sowing to the trilobal period. N_GS_ is the number of green stalks per m^−2^, N_MS_ is the number of green main stems per m^−2^, and N_T_ is the number of green tillers per m^−2^.

### 2.4 Biomass and phytohormone contents in tiller nodes at pre-winter stage

To determine the biomass according to the tillering survey time described above, another row with uniform growth was selected, and 10 plants were collected consecutively in each repeated plot. The roots were removed, and the tillers were separated from the main stems. The treated samples were dried at 120°C for 20 min and then dried to a constant weight. The biomass (g m^−2^) of minimum stems and tillers at different tillering positions was measured. Five plants with representative growth potentials were collected from each plot during the sampling period, and the tiller nodes were cut and mixed (**Figure 4A**). Each repeated plot was used as a mixed sample and stored at −80°C.

The SL content in the tillering node was determined using high-performance liquid chromatography-mass spectrometry (Liu et al., 2011) on a TSQ Quantum triple quadrupole mass spectrometer and UltiMate 3000 RS chromatograph. Fresh wheat tiller buds (0.5 g) were mixed with 1 ml of pure methanol and zirconia grinding beads and ground for 5 min in liquid nitrogen. The supernatant was obtained by centrifugation at 8,000×*g* at 4°C for 10 min. The supernatant was filtered through a 0.22 μm membrane and used for analysis. SL chromatogram collection and integration were performed using Xcalibur software (version 3.0; Thermo Fisher Scientific, Waltham, MA, USA).

To determine the contents of IAA, zeatin riboside (ZR), and gibberellin (GA), 1.0 g of fresh wheat tiller bud was placed in a pre-cooled mortar, mixed with 6 ml of 80% pre-cooled methanol, ground in an ice bath, and extracted at 4.0 °C for 20 h. After centrifugation at 8,000×*g* at 4 °C for 10 min, the supernatant was collected to determine the IAA, GA, and ZR contents in the tillering node using an Agilent 1100 high-performance liquid chromatograph (Agilent, Santa Clara, CA, USA) (Liu et al., 2021a; Liu et al., 2022; Mao et al., 2022).

### 2.5 Percentage of effective tillers and grain yield

To determine the percentage of effective tillers, we counted all effective spikes (ES, kernels per spike ≥5) in the ripening stage (BBCH 89) in the sample segments marked as “BSN and tillers” and calculated the number of effective tillers in the overwintering stage (ET) and spring (EP) based on the color of nylon rope added to the tillers formed during different seasons. The percentage of ET at different growth stages was calculated using Equation 4.


Equation 4 $$\text{ETgs}\left(\%\right)=\frac{\text{ESgs }\left(\times 10^{4}{\text{ hm}}^{-2}\right)}{\text{Tgs }\left(\times 10^{4}{\text{ hm}}^{-2}\right)}\times 100$$

ETgs is the effective tiller percentage at a specific growth stage, and ESgs and Tgs are the effective tillers and maximum tiller numbers at a specific growth stage.

Representative growth sample points (1.0 m length × 1.5 m width) were selected from each plot for GY measurements during the ripening stage (BBCH 89), calculated spikes per hectare, and then selected 20 randomly from all of them to count the grain number per spike. GY were calculated using Equation 5 after drying the samples to a standard moisture content of 12.5%.


Equation 5 $$\text{GY}\;\left({\text{kg hm}}^{-2}\right)=\frac{\text{ESW }\times \text{ GN }\times \text{ GW }\times \;0.85}{100}\;$$

where GY is the grain yield, ESW is the effective spike number during the entire growth period (×10^4^ spikes hm^−2^), GN is the kernel number per spike (kernels spike^−1^), GW is the 1,000-grain weight (g), and 0.85 is an empirical coefficient.

### 2.6 Path analysis of tillering characteristics and yield factors

Wheat GY was considered the dependent variable, and 14 factors affecting its formation were considered independent variables (*X_i_*): TP, TS, total tillers (TT), effective tillers in the pre-winter stage, effective tillers in spring, total effective tillers, effective spikes on the main stem, percentage of effective tillers in the pre-winter stage, percentage of effective tillers in spring, percentage of total effective tillers throughout the growth period, percentage of main stem spikes to total effective spikes, ESW, kernel number per spike (KN), and GW, abbreviated as *X_1_*–*X_14_*, respectively. After linear stepwise regression, *X_i,_* with a partial regression coefficient of<0.05, was included in the regression model. The effect of *X_i_* on GY is expressed as the direct path coefficient (DDC). The indirect effect of *X_i_* on *GY* through *X_i_* was expressed as the indirect path coefficient (IDC), calculated using Equation 6.


Equation 6 $$IDC_{ijGY}=r_{ij}\times DDC_{jGY}$$

where IDC*_ijGY_* is the IDC of *X*_i_ to *GY* through *X_j_* (*X_i_*_→_ *_jZ_*);*r_ij_* is the Pearson correlation between *X*_i_ and *X_j_*; and DDC*_jGY_* is the direct path coefficient of *X_j_* to GY.

### 2.7 Data processing and statistical analysis

Analysis of variance (ANOVA) was performed using SPSS 26.0 (IBM, NY, USA). Data are presented as the mean ± standard error (SE). A one-way ANOVA was conducted, and the Student’s t-test was used to compare treatment means at the 5% level. Pearson correlation analysis and linear regression were used for correlation and path analysis using SPSS software (version 26.0).

## 3 Results

### 3.1 Optimizing plant spatial competition increases tillers number and biomass accumulation of winter wheat

#### 3.1.1 Tillers number

**Figure 5:**
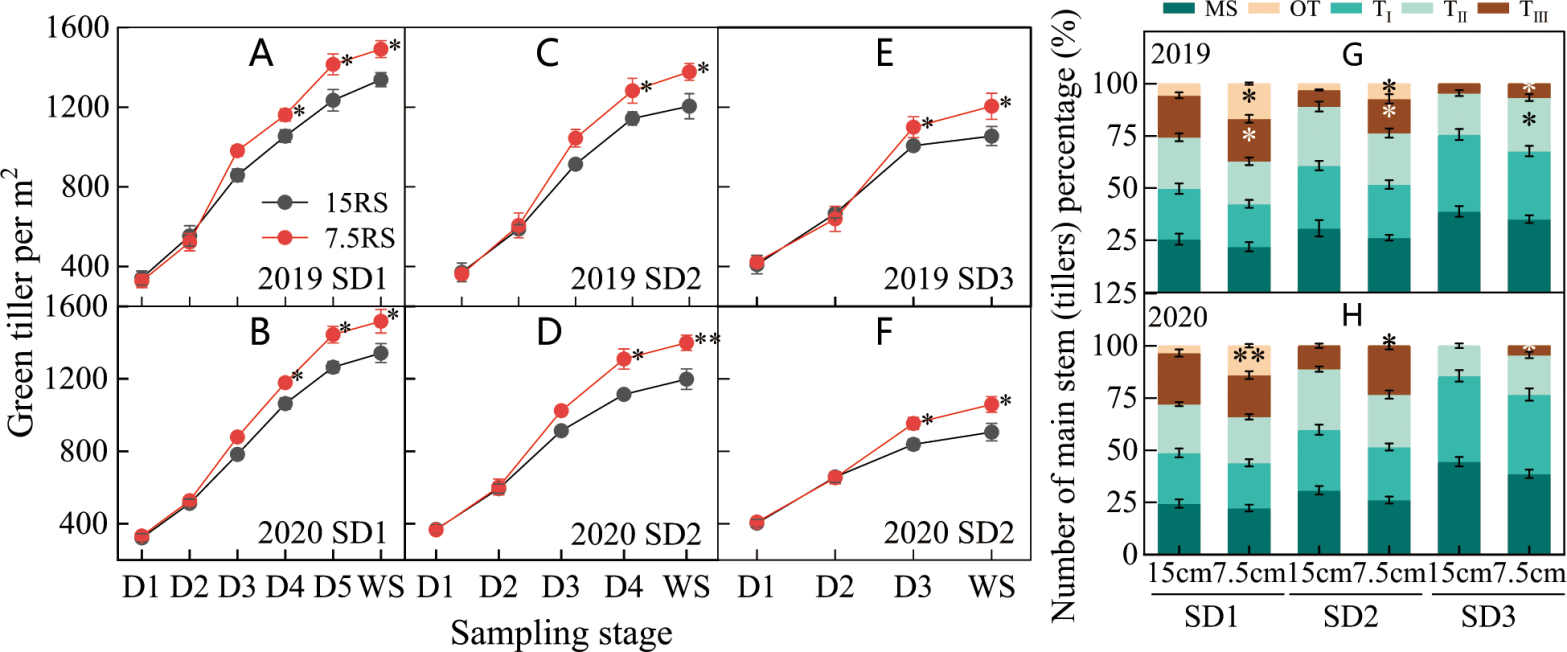
Number of wheat tillers at pre-winter stage under different sowing dates and row spacings. **(A, C, E)** show the green tillers number for wheat in pre-winter stage for SD1, SD2 and SD3 in 2019, respectively. **(B, D, F)** show the green tillers number for wheat in pre-winter stage of SD1, SD2 and SD3 in 2020, respectively. **(G, H)** show the quantity proportion of main stems and tillers for wheat in 2019 and 2020, respectively. “*, **” indicate significant difference at 0.05 level. **P<0.01; *P<0.05. Significant markers represent the differences between 7.5 cm and 15 cm row spacing treatments at the same sowing date and observation period. Number of wheat tillers at pre-winter stage under different sowing dates and row spacings.

Compared with those from the 15RS group, tillers from the 7.5RS group increased in the pre-winter stage for the SD1, SD2, and SD3 groups by 12.3%, 15.5%, and 15.6%, respectively, until the overwintering stage (annual average value over two years). There was no significant difference in the number of tillers between SD1 and SD2 sowing dates from D1 (trilobal stage) to D3. At the beginning of the D4 stage, the promotion effect of LSRE on tillering became obvious, and the number of 7.5RS tillers began to be significantly higher than that of 15RS, and the difference reached a maximum at the WS. LSRE began to promote tillering at the beginning of the D4 stage significantly, and the tiller number of 7.5RS started to become significantly higher than that of 15RS, and the difference reached a maximum until the overwintering stage. Under SD3 treatment, the tiller number differed between the two-row spacing treatments at the D3 stage; 7.5RS was significantly higher than 15RS, and the difference reached a maximum until WS (**Figures 5A–F**).

Analysis of the stem composition under different row spacings before the overwintering stage showed that following LSRE treatment, the percentage of the third, fourth, and other tiller numbers of the main wheat stem at the SD1 sowing date and the percentage of the third level of the main stem and other tiller numbers at the SD2 sowing date increased. The average increase for the three sowing treatments was 9.8 percentage points (2-year average) (**Figures 5G, H**).

#### 3.1.2 Tiller biomass accumulation

**Figure 6:**
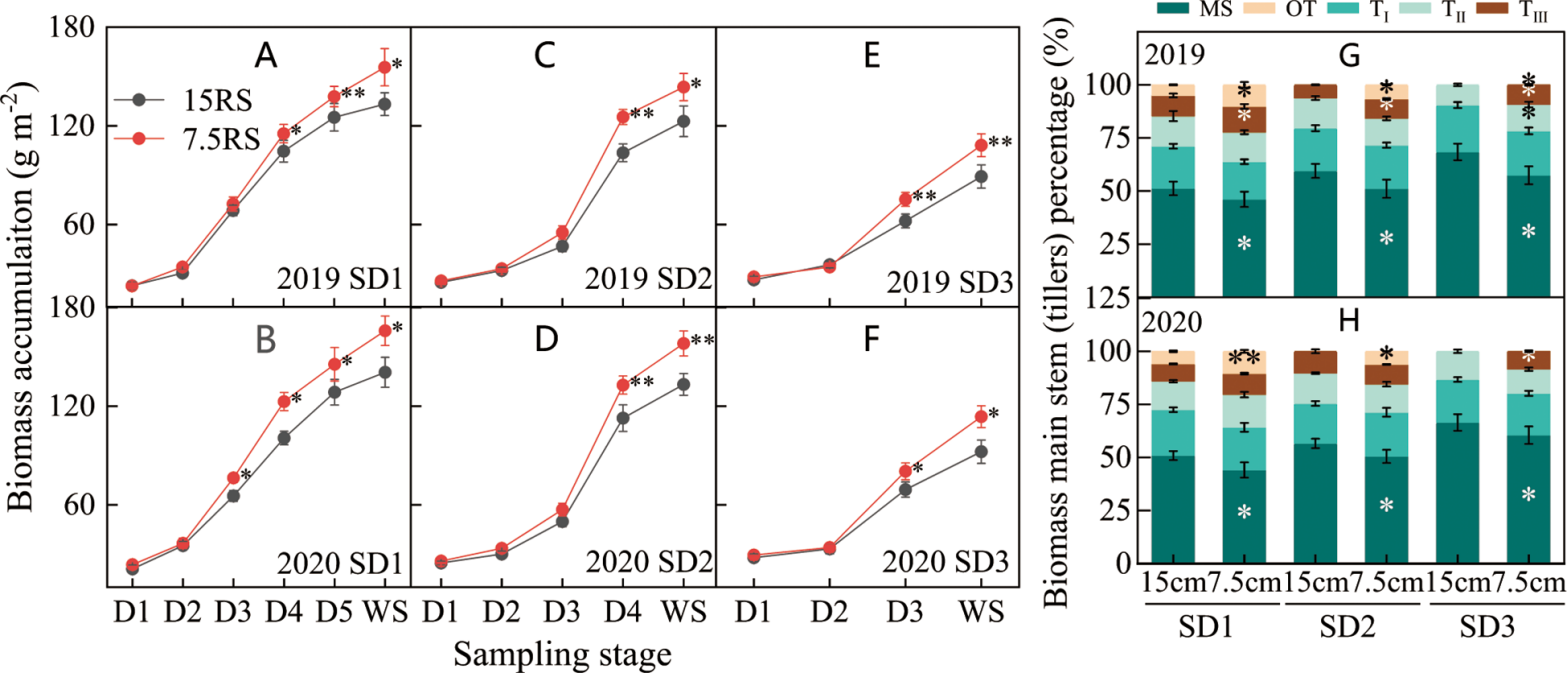
Biomass of wheat tillers at pre-winter stage under different sowing dates and row spacings. **(A, C, E)** show the dry matter accumulation for wheat in pre-winter stage for SD1, SD2 and SD3 in 2019, respectively. **(B, D, F)** show the dry matter accumulation for wheat in pre-winter stage of SD1, SD2 and SD3 in 2020, respectively. **(G, H)** show the biomass accumulation proportion of main stems and tillers for wheat in 2019 and 2020, respectively. ”*, ** ”indicate significant difference at 0.05 level. **P<0.01; *P<0.05. Significant markers represent the differences between 7.5 cm and 15 cm row spacing treatments at the same sowing date and observation period. MS stands for main stem, T_I,_ T_II_, and T_III_ represent the first, second, and third tillers of the main stem respectively, and OT represents the fourth and other tillers of the main stem.

From the trilobal stage (D1) to the overwintering stage (WS) during SD1 and SD2, the tiller biomass of all treatments increased slowly and rapidly from D1 to D2 and D2 to D4, respectively (**Figures 6A–D**). The difference between 7.5RS and 15RS was slight from D1 to D2 and then gradually increased; 7.5RS showed a faster biomass accumulation rate. At WS, the biomass accumulation difference between the two-row spacing groups reached a maximum (**Figures 6A–F**). The biomass of 7.5RS tillers in the three sowing-date groups increased by 20.9% compared to that of 15RS tillers (2-year average). In addition, the difference in biomass accumulation between the two-row spacings increased with delays in the sowing date: compared with biomass accumulation in the 15RS wheat field, that of the 7.5RS field under SD1 treatment increased by 17.9%, SD2 treatment increased by 18.7%, and SD3 treatment increased by 23.1% (2-year annual average). In the winter stage (**Figures 6G, H**), the proportion of 7.5RS main stem biomass in plants for each sowing date was significantly lower than that of 15RS, with SD1, SD2, and SD3 reduced by 6.0%, 7.3%, and 8.5%, respectively (2-year average). The proportion of biomass of the third tiller and other tillers (fourth tiller and second tiller on the main stem) was significantly higher than that of the 15RS treatment, with an average decrease of 5.7% (2-year average).

#### 3.1.3 Accumulated temperature of tillers

**Figure 7:**
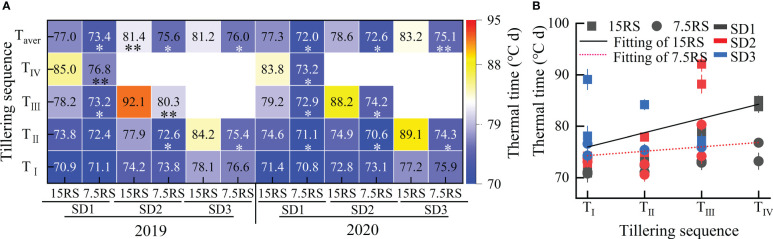
Accumulated temperature required for tillering at different tillers of wheat at pre-winter stage under different sowing dates and row spacings. **(A)** shows the AT required for different treatments of tillers at each level for wheat. **(B)** shows the linear fitting of AT required for wheat tillers with 7.5RS and 15RS. “*, ** ”indicate significant difference at 0.05 level. ** *P<*0.01; *P<0.05. Significant markers represent the differences between 7.5 cm and 15 cm row spacing treatments at the same sowing date and observation period. T_I_, T_II_, T_III_, and T_IV_ represent the first, second, and third tillers of the main stem, respectively. T_aver_ represents the average of T_I_, T_II_, T_III_, and T_IV_. 15RS and 7.5RS fitting equations are *Y*_15RS_ = +70.66+3.78*x* and *Y*_7.5RS_ = 73.3 + 0.55*x*, respectively.

As shown in **Figure 7A**, for the two-row spacing treatments, the AT required for the formation of the first tiller (T_I_) on the main stem of wheat for the three sowing-date groups did not differ significantly between the two-row spacing treatments, whereas that for forming the second tiller (T_II_) differed. The AT required for SD2 and SD3 for 7.5RS was significantly lower than that for 15RS, with average decreases of 6.3% and 13.5%, respectively (2-year average). Compared with the AT required for forming higher tillers, LSRE promoted tillering. The AT required for forming the third and fourth tillers of the main stem at SD1 decreased by 7.2% and 11.2% for 7.5RS compared with those at 15RS, respectively, in the two test years.

Overall, LSRE reduced the AT required for wheat tillering in the pre-winter stage by 5.7, 7.4, and 8.0% for SD1, SD2, and SD3, respectively (2-year average). Furthermore, according to the linear fitting of the AT required for tillering in the different row spacing treatments, with the postponement of the tillering sequence, the AT required for 15RS tillering significantly increased by more than 7.5RS. The slopes of the fitting equations were 3.78 and 0.55 for 15RS and 7.5RS, respectively (**Figure 7B**). Under the same row spacing, the AT required for tillering at the same tiller position was significantly increased when the sowing date was delayed.

### 3.2 Optimizing plant spatial competition can change phytohormone content in tillering node of winter wheat

#### 3.2.1 Contents of IAA and ZR

**Figure 8:**
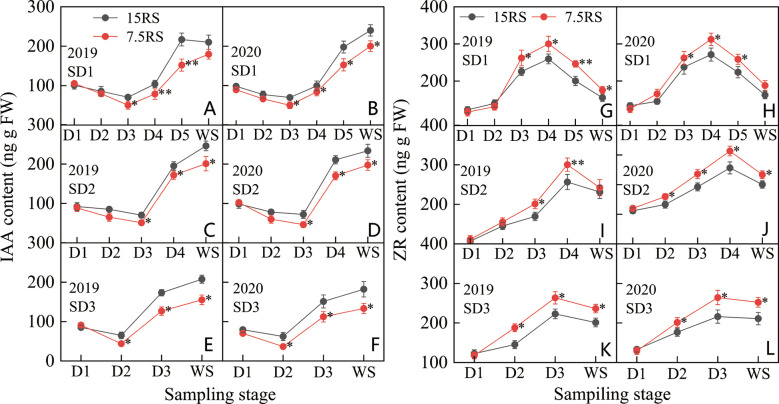
IAA and ZR content of wheat tillers at pre-winter stage under different sowing dates and row spacings. **(A, C, E)** show the IAA content for wheat in pre-winter stage for SD1, SD2 and SD3 in 2019, respectively. **(B, D, F)** show the IAA content for wheat in pre-winter stage of SD1, SD2 and SD3 in 2020, respectively. **(G, I, K)** show the ZR content for wheat in pre-winter stage for SD1, SD2 and SD3 in 2019, respectively. **(H, J, L)** show the ZR content for wheat in pre-winter stage of SD1, SD2 and SD3 in 2020, respectively. “*, **” indicate significant difference at 0.05 level. **P<0.01; *P<0.05. Significant markers represent the differences between 7.5 cm and 15 cm row spacing treatments at the same sowing date and observation period.

The IAA content of the wheat tillering nodes first decreased and then increased in each treatment from the trilobal stage (D1) to WS. The minimum IAA content during SD1 and SD2 was at the D3 stage, but the lowest IAA content during SD3 was at the D2 stage. At SD1 and SD2, the IAA content in the 15RS treatment decreased slightly (27.4%) at the D1–D3 stages, whereas that of the 7.5RS treatment decreased significantly (48.3%). After D3, the IAA content of the two-row spacing treatments increased rapidly, although 7.5RS remained lower than 15RS (20.0% lower than the 2-year average). For SD3, in the D1–D2 stages, the IAA content of the 7.5RS treatment decreased more significantly by 28.2%, and its decline was higher than that of 15RS (2-year average). During D2–WS, the IAA content of the two-row spacing groups began to increase. However, the growth rate of 7.5RS was much lower, at 52.7% lower than 15RS on average (**Figures 8A–F**).

As shown in **Figures 8G–L**, the change in the ZR content in D1–WS for the three sowing-date groups was opposite to that of the IAA content; the ZR content first increased and then decreased. The highest ZR content in SD1 and SD2 was observed at the D4 stage, whereas that in SD3 was at the D3 stage. The peak value of ZR content for the three sowing-date groups was 19.9% higher for 7.5RS than for 15RS over two years. After the peak value, the ZR content of all treatments decreased rapidly; however, in the overwintering period, the ZR content of 7.5RS remained significantly higher than that of 15RS by 15.4%, 15.3%, and 19.4% for SD1, SD2, and SD3, respectively. After the peak ZR content was reached in each treatment group, 7.5RS showed a more significant decrease; the average decrease for the three sowing-date groups was 4.0% higher than that for 15RS (2-year average). Overall, in the pre-winter stage of winter wheat, lower IAA and higher ZR content were more conducive to the generation of wheat tillers. The IAA content of 7.5RS in the three sowing-date groups was 19.4% lower than that of 15RS, whereas the ZR content was 13.1% higher (2-year average).

#### 3.2.2 Contents of SLs and GA

**Figure 9:**
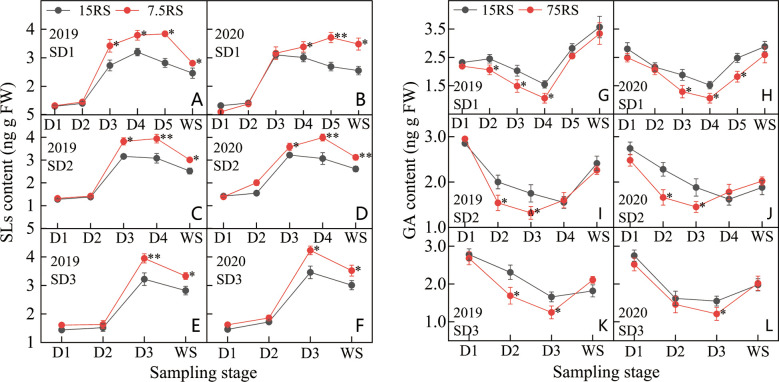
SL and GA content of wheat tillers at pre-winter stage under different sowing dates and row spacings. **(A, C, E)** show the SLs content for wheat in pre-winter stage for SD1, SD2 and SD3 in 2019, respectively. **(B, D, F)** show the SLs content for wheat in pre-winter stage of SD1, SD2 and SD3 in 2020, respectively. **(G, I, K)** show the GA content for wheat in pre-winter stage for SD1, SD2 and SD3 in 2019, respectively. **(H, J, L)**show the GA content for wheat in pre-winter stage of SD1, SD2 and SD3 in 2020, respectively. “*, **” indicate significant difference at 0.05 level. **P<0.01; *P<0.05. Significant markers represent the differences between 7.5 cm and 15 cm row spacing treatments at the same sowing date and observation period.

As shown in **Figures 9A–F**, the SL content in the tillering node of wheat under 7.5RS treatment was significantly higher than that under 15RS treatment (25.9% higher in the two years) at the D4 to WS stage of SD1; the maximum difference was observed at the D4 stage (37.9% higher in the two years). At the SD2 sowing date, the difference in SL content between the 7.5RS and 15RS treatments was like that at the SD1 sowing date, demonstrating that the difference was small before the D2 stage and large after the D2 stage. Additionally, after the D4 stage, the SL content in the 7.5RS treatment was significantly higher than that in the 15RS treatment (31.5% higher than that in the two years), with a peak difference of 21.5%. During the D3–WS stage of SD3 treatment, the SL content in the 7.5RS treatment was 20.0% higher than that in the 15RS treatment, with the largest difference observed in the D3 stage; 7.5RS was 22.5% higher than that in the 15RS. Overall, from the D1 to WS stage, the SL content in the wheat tiller nodes of the two-row spacing treatments for the three sowing-date groups first increased and then decreased; the LSRE treatment increased the SL content in the wheat tiller nodes by 17.5% in the two years. In addition, the SL content increased when the sowing date was delayed.

At the beginning of the D1 stage, the GA content in the tiller nodes of all treatments first decreased and then increased during the growth period. At SD1, the minimum GA content was observed at the D4 stage, whereas the minimum GA content of SD2 and SD3 appeared at the D3 stage (**Figures 9G–L**). Compared with the two-row spacing treatments, the GA content decreased from the trilobal stage to a minimum value, with SD1, SD2, and SD3 showing 40.5%, 1.0%, and 25.8% lower values in 7.5RS than in 15RS, respectively. The GA content of 7.5RS was significantly lower than that of 15RS for SD1 from D2 to D4, SD2 from D2 to D3, and SD3 from D3. For the different sowing dates, the lowest GA content tended to increase with a delay in the sowing date; the increase in 7.5RS was more obvious than that in 15RS. From SD1 to SD3, the lowest GA content of 7.5RS increased by 16.3%, whereas that of 15RS increased by only 4.6%. There was no significant difference in GA content between the two rows at each sowing date until the overwintering stage. LSRE reduced GA content, with decreases of 15.7%, 9.1%, and 9.3% for SD1, SD2, and SD3, respectively.

#### 3.2.3 IAA/ZR and SLs/GA

**Figure 10:**
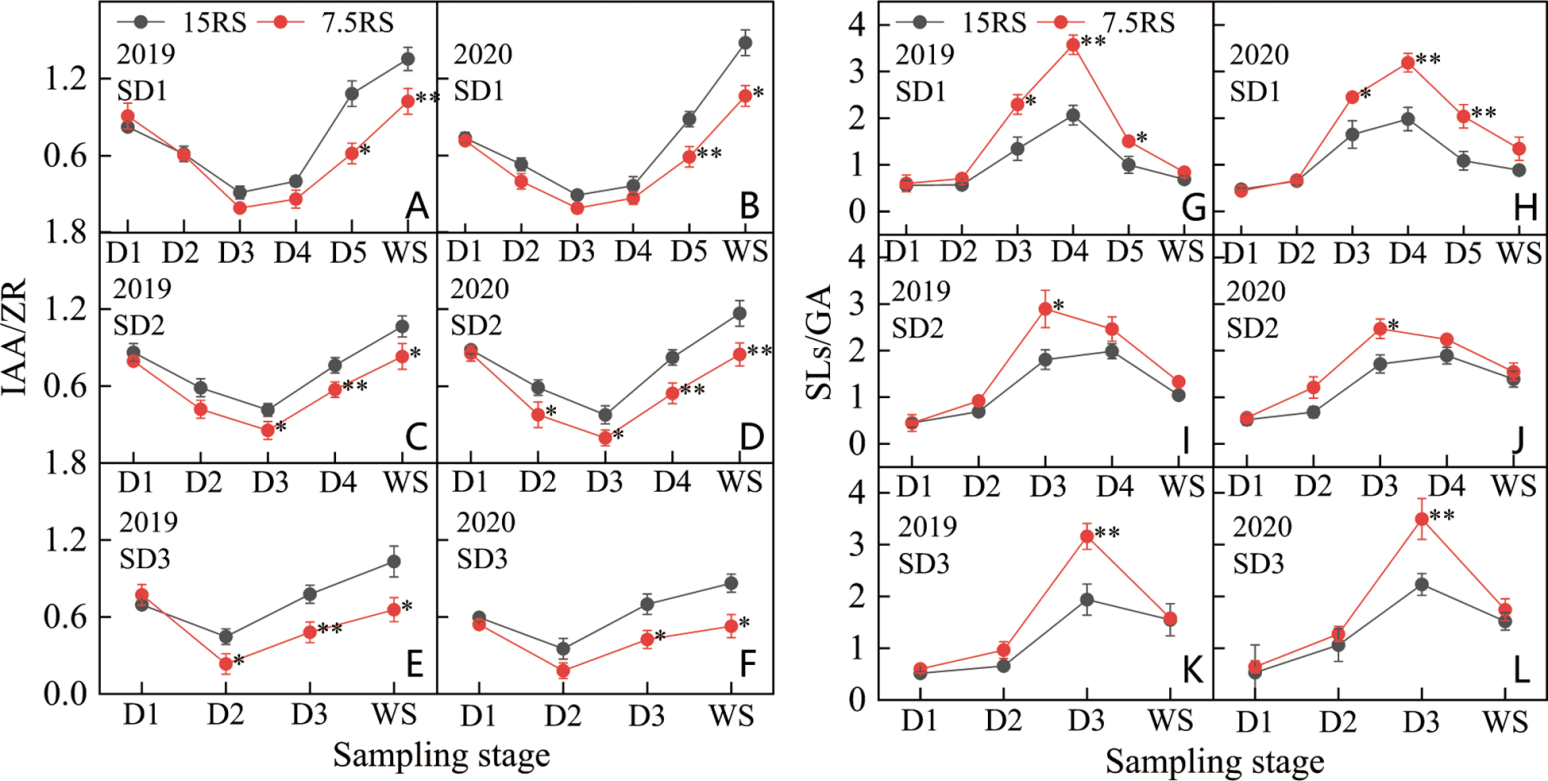
IAA/ZRand SLs/GA of wheat tillers at pre-winter stage under different sowing dates and row spacings. **(A, C, E)** show the IAA/ZR for wheat in pre-winter stage for SD1, SD2 and SD3 in 2019, respectively. **(B, D, F)** show the IAA/ZR for wheat in pre-winter stage of SD1, SD2 and SD3 in 2020, respectively. **(G, I, K)** show the SLs/GA for wheat in pre-winter stage for SD1, SD2 and SD3 in 2019, respectively. **(H, J, L)** show the SLs/GA for wheat in pre-winter stage of SD1, SD2 and SD3 in 2020, respectively. “*, **” indicate significant difference at 0.05 level. **P<0.01; *P<0.05. Significant markers represent the differences between 7.5 cm and 15 cm row spacing treatments at the same sowing date and observation period.

IAA/ZR (I/Z) decreased and then increased from the trilobal stage (D1) to WS. For SD1 and SD2, the minimum I/Z value appeared at the D3 stage, but the minimum value during SD3 appeared earlier (D2) (**Figures 10A–F**). At the lowest I/Z point, the 7.5RS group was lower than the 15RS group by 36.7%, 43.0%, and 48.0% for SD1, SD2, and SD3, respectively (2-year average). After the lowest point, the I/Z of each treatment began to increase rapidly, but the increase in 7.5RS was lower than that in 15RS, and the I/Z increase of 7.5RS was 35.7% lower than that of 15RS until the WS. The I/Z of 7.5RS treatment was significantly lower than that of 15RS at the beginning of the SD1 D5 stage and SD2 and SD3 D3 stages in WS, with decreases of 31.4%, 29.5%, and 35%, respectively (2-year average). Overall, reducing row spacing could reduce the I/Z of wheat tiller nodes during the pre-winter stage. The 7.5RS group showed 22.9%, 24.4%, and 30.1% lower values than the 15RS group at SD1, SD2, and SD3, respectively, with an average decrease of 25.8%.

As shown in **Figures 10G–L**, the SLs/GA (S/G) of D1-WS and each treatment first increased and then decreased. From D1 to D2, the S/G of the two-row spacing treatments increased slightly and showed no significant differences in the three sowing-date groups. At the beginning of the D2 stage, the S/G values of all treatments increased significantly. Except for SD1 in 2020, the peak value of the other treatments was observed in the D3 stage, with 7.5RS showing a higher value than 15RS. After D3, SD1 treatment continued to increase in 2020 and reached a peak in D4. The S/G of 7.5RS remained significantly higher than that of 15RS. In 2020, in addition to SD1, the S/G of the other sowing date treatments decreased to varying degrees from D3 to WS, whereas 7.5RS further decreased. There was no significant difference in the S/G between the two-row spacing treatments until WS. Overall, reducing row spacing improved the S/G of the tillering node of wheat in the pre-winter stage, and SD1, SD2, and SD3 led to increases of 51.7%, 32.1%, and 33.9%, respectively. A lower I/Z ratio and a higher S/G ratio significantly promote wheat tillering. In particular, under late sowing conditions, the lower I/Z and higher S/G that appeared earlier after the trilobal stage can promote tillering under lower temperatures at the tillering stage, thus achieving a sufficient population.

### 3.3 Correlation between endogenous phytohormone content and tillering characteristics under optimal plant distribution of winter wheat

**Figure 11:**
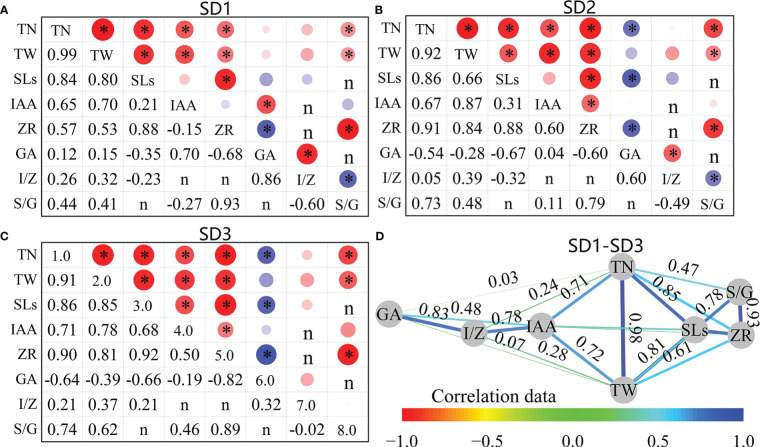
Correlation between tiller number, biomass and phytohormone contents of wheat tillers at pre-winter stage under different sowing dates and row spacings. **(A–C)** show the correlation data of SD1, SD2 and SD3, respectively. (D) shows the average correlation data of SD1-SD3. “*r*” is Person two tailed correlation coefficient, “*” indicate significant difference at 0.05 level. *P<0.05. **(D)** Figure D is the network diagram of the correlation coefficient adjacency matrix between indicators, and the node layout adopts the Kamada–Kawai algorithm. The connection represents *r* between each two nodes. The thicker the connection, the greater the absolute value of *r*, and the data of **(D)** comes from the mean of SD1, SD2, and SD3.

As shown in **Figures 11A–C**, the number of tillers (TP) in the pre-winter stage was significantly positively correlated with SLs, IAA, and ZR at the tiller nodes for the three sowing-date groups (*P*<0.05), and the average correlation coefficients (*r*) were 0.85, 0.68, and 0.79, respectively. Among them, the correlation between the TN and SLs of the SD1 treatment was the highest (*r* = 0.84), whereas that between the SD2 and SD3 treatments was the highest (*r* = 0.91 and 0.90, respectively). Tiller biomass (TW) was also significantly positively correlated with SLs, IAA, and ZR in the three sowing-date groups (*P<*0.05), with average correlation coefficients of 0.77, 0.78, and 0.73, respectively (**Figure 11D**). The TW of SD1 and SD3 showed the highest correlation coefficient with SLs (*r* = 0.8 and 0.85, respectively), whereas the TW of SD2 showed the highest correlation coefficient with IAA (*r* = 0.87). The TP and GA of SD2 and SD3 were significantly negatively correlated, whereas SD1 showed no significant correlation. There was also a strong correlation between tiller growth and hormone content; for example, there was a significant correlation between TP, TW, and S/G (*r* = 0.64 and 0.50).

The contents and ratios of different hormones were correlated at the tillering stage before winter. SLs in the three sowing-date groups were significantly positively correlated with ZR (*r* = 0.89) and significantly positively correlated with IAA only at SD3 (*r* = 0.68). SLs were negatively correlated with GA in all three sowing-date groups, and SD2 and SD3 were significantly correlated (*P<*0.05). IAA and ZR showed significant positive correlations with SD2 and SD3 (*r* = 0.55) but exhibited no significant correlation with SD1 (*P >*0.05). The ZR and GA of the three sowing-date groups were negatively correlated (mean *r* = 0.70). Further analysis of the correlation between tiller growth characteristics and various hormones at different sowing dates showed that when the sowing date was postponed, the correlation between TP and TA, TW and IAA, and ZR and S/G showed an increasing trend. In contrast, the correlation between TP and GA changed from insignificant at suitable sowing dates to a significant negative correlation with delayed sowing dates. TN and TW were significantly and positively correlated with SLs, IAA, ZR, and SLs/GA, but not with IAA/ZR for the three sowing-date groups, and TP and GA were significantly negatively correlated when the sowing date was delayed.

### 3.4 Optimizing plant spatial distribution increased the number of effective tillers and yield of winter wheat

#### 3.4.1 Number of effective tillers

As shown in **Table 2**, when the year (Y) was used as a single source of variation, the number of tillering panicles (ETP and ETS) and the ratio of effective tillers (PETP and PETS) were significantly different (*P*<0.01), with no significant differences in the other indicators. Comparing different RS and SD, except for the lack of a significant difference between different row spacings for BSN and no significant difference for ETT between different SD, other indicators did not significantly differ when row spacing and SD were the single sources of variation. Further analysis of the interaction between different sources of variance revealed significant differences in the Y × SD interaction, except for those in BSN and ETT. The indicators showed no significant differences in the Y × RS interaction. Under the SD × RS interaction, TS and PETS showed significant and very significant differences, respectively, whereas the other indices showed no significant difference. However, under the interaction of factors, such as Y × SD × RS, as the source of variation for different tillering characteristics, only ETS and PETS showed highly significant differences, whereas the other indicators showed no significant differences.

**Table 2:** Characteristics of tillering and spike formation of wheat under different sowing dates and row spacings.

Year	SD	RS (cm)	BSN (×10^4^/hm^2^)	Number of tillers (×10^4^ hm^2^)			Effective tillers number (×10^4^ hm^2^)			Percentage of effective tillers (%)		
				TP	TS	TT	ETP	ETS	ETT	PETP	PETS	PETT
2019–2020	SD1	15	342.0	921.0	340.8	1261.8	372.0	75.0	447.0	40.4	22.1	35.5
		7.5	327.0	1156.5**	324.0	1480.5**	411.0*	60.0**	471.0	35.7*	18.5**	31.9*
	SD2	15	370.5	772.5	460.8	1233.3	313.5	79.5	393.0	40.4	17.2	31.8
		7.5	361.5	1015.5**	426.0	1441.5**	363.0	43.5**	406.5	35.7	10.2**	28.2*
	SD3	15	409.5	597.0	399.6	996.6	192.0	94.5	286.5	32.1	23.6	28.7
		7.5	423.0	781.5**	424.8	1206.3**	240.0*	60.0**	300.0	30.7	14.1**	24.9*
2020–2021	SD1	15	328.5	967.5	386.4	1353.9	369.0	88.5	457.5	38.2	22.9	33.8
		7.5	339.0	1227.0**	315.6*	1542.6**	405.0	51.0**	456.0	33.0*	16.1**	29.6*
	SD2	15	369.0	849.0	415.2	1264.2	318.0	64.5	382.5	37.5	15.5	30.3
		7.5	366.0	1045.5**	381.6	1427.1**	367.5**	36.0**	403.5	35.1	9.4**	28.3
	SD3	15	403.5	502.5	412.8	915.3	265.5	54.0	319.5	53.0	13.1	35.0
		7.5	409.5	709.5**	404.4	1113.9**	304.5	25.5**	330.0	43.3*	6.3**	29.8*
Year (Y)			ns	ns	ns	ns	**	**	ns	**	**	ns
Sowing date (SD)			**	**	**	**	**	**	**	*	**	**
Row space (RS)			ns	**	**	**	**	**	ns	**	**	**
Y×SD			ns	**	**	**	**	**	ns	**	**	**
Y×RS			ns	ns	ns	ns	ns	ns	ns	ns	ns	ns
SD×RS			ns	ns	*	ns	ns	ns	ns	ns	**	ns
Y×SD×RS			ns	ns	ns	ns	ns	**	ns	ns	**	ns

RS and BSN represent row space and basic seedling number. TP, TS, and TT represent tillers number in pre-winter stage, spring, and the whole growth period, respectively. ETP, ETS and ETT represent effective tillers in pre-winter stage, spring, and the whole growth period, respectively. PETP, PETS and PETT represent the percentage of effective tillers in pre-winter stage, spring, and the whole growth period, respectively. “*, **” indicate significant difference at 0.05 level. **P<0.01; *P<0.05, ns, not statistically significant at *P*<0.05.

LSRE treatment had no significant effect on BSN but significantly increased TP and TT by 29.9% and 17.3%, respectively, for the three sowing-date groups (2-year average). Although the TS of wheat treated with LSRE decreased, the difference was not statistically significant. LSRE increased the number of tillers and spikes by 3.7% for the three sowing-date groups, and ETP increased significantly by 15.2% for the three sowing-date groups (2-year average). LSRE also significantly inhibited ETS by 40.2% (2-year average) in the three sowing-date groups. The percentage of tillers and spikes at each stage of the LSRE treatment decreased by 5.0% (average of three sowing-date groups in two years).

Overall, the interaction of RS, SD, and Y × SD as sources of variation significantly affected the tillering and tiller panicle formation characteristics, whereas Y, as a single source of variation, significantly impacted ETP, ETS, PETP, and PETS.

#### 3.4.2 Grain yield factors

Analysis of the interaction effects (**Table 3**) showed that the kernel number per spike (KN) and 1,000 grain weight (GW) significantly differed between years (*P*<0.01), whereas the ESW and GY showed no significant differences (*P >*0.05). Except for KN, the SD and row spacing significantly affected the other two yield factors (GW and ESW). The single effects of yield on Y, SD, and RS were significant (*P<*0.05) or highly significant (*P<*0.01). The reduction in row spacing tended to increase ESW and KN, and the average heights of plants from the three sowing-date groups were 1.9% and 0.4%, respectively, but the differences were not significant. In addition, after delaying the sowing date, the LSRE treatment had a more noticeable effect on the ESW increase. The LSRE treatment improved GW by 4.7% and 3.2% in 2020 and 2021, respectively. SD1 and SD2 in 2020 showed significant and highly significant differences, respectively, whereas no significant differences were observed for the other sowing dates. The LSRE treatment significantly increased yield by 6.8% and 5.7% in 2020 and 2021, respectively. However, the yield increase effect of the different sowing dates differed between the two trial years. In 2020, the yield increase effect of LSRE on a suitable sowing date was significant (7.6%), whereas in 2021, the yield increase effect of SD2 was significant (6.3%). LSRE can improve ESW and KN to some extent and significantly improve GW, although most treatments lead to no significant difference. LSRE significantly increased yield by 6.3% for the three sowing-date groups.

**Table 3:** Grain yield factors of wheat under different sowing dates and row spacings.

Sowing date	Row space (cm)	2019–2020				2020–2021			
		ESW km^−2^ (×10^−4^)	KN	GW (g)	GY (kg hm^−2^)	ESW km^−2^ (×10^−4^)	KN	GW (g)	GY (kg hm^−2^)
SD1	15	789.0	32.6	48.1	10,510.8	786.0	35.8	45.8	10,964.5
SD1	7.5	798.0	33.1	50.4*	11,311.1*	795.0	36.0	47.3	11,509.8*
SD2	15	763.5	33.3	49.0	10,592.0	751.5	35.8	46.6	10,651.7
SD2	7.5	768.0	32.6	52.8**	11,238.9*	769.5	36.1	48.0	11,322.6**
SD3	15	696.0	32.2	52.2	9,941.4	723.0	34.2	48.7	10,229.0
SD3	7.5	723.0	32.6	53.0	1,0615.0*	739.5*	34.3	50.3	10,836.4**
Year (Y)		ns	**	**	ns	ns	**	**	ns
Sowing date (SD)		**	**	**	**	**	**	**	**
Row space (RS)		**	ns	**	**	**	ns	**	**
Y × SD		ns	ns	ns	ns	ns	ns	ns	ns
Y × RS		ns	ns	ns	ns	ns	ns	ns	ns
SD × RS		ns	ns	ns	ns	ns	ns	ns	ns
Y × SD × RS		ns	ns	ns	ns	ns	ns	ns	ns

ESW, KN, GW, and GY represent total effective spikes in the whole growth period, kernel number per spike, 1,000-grain weight, and grain yield, respectively. “*, **” indicate significant difference at 0.05 level. **P<0.01; *P<0.05, ns, not statistically significant at *P*<0.05.

### 3.5 Optimizing plant spatial distribution changes the contribution of tillers and yield factors to yield

**Figure 12:**
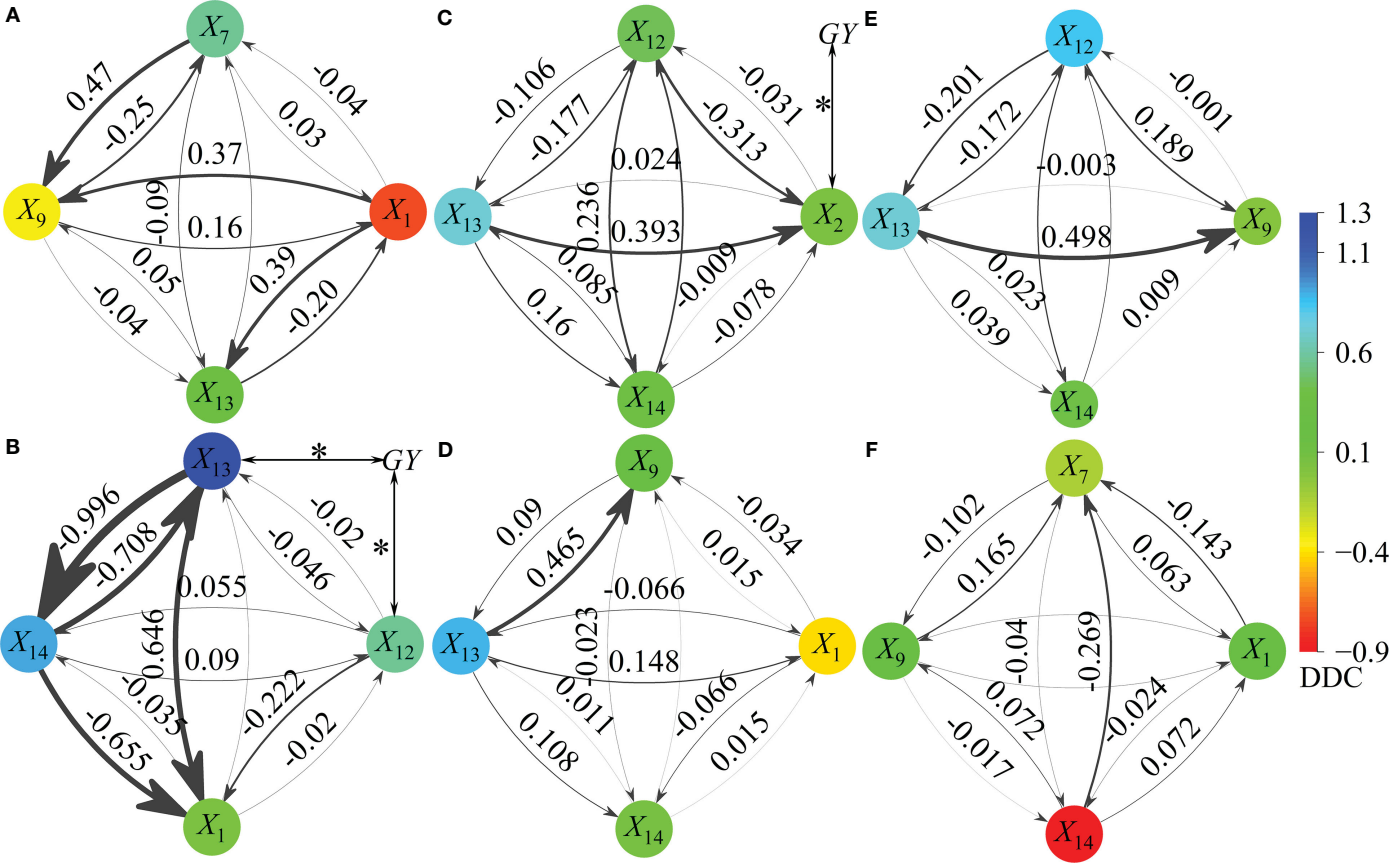
Path effect of different action factors (independent variables) on grain yield (dependent variables). *X_1_*, *X_2_*, *X_7_*, *X_9_*, *X*_12_, *X*_13_, *X*_14_, and GY represent tillers in pre-winter stage (TP), tillers in spring stage (TS), effective spikes on main stem (ESM), percentage of effective tillers in spring (PESS), effective spikes in the whole growth period (ESW), kernel number per spike (KN), 1,000-grain weight (GW) and grain yield (GY) respectively. ANOVA: *P <0.05, it is the Pearson’s two tailed correlation coefficient between action factor (*X_i_*) and the GY. The arrow points to the effect of one factor on GY through another factor, and the thicker the line, the greater the indirect path coefficient (absolute value of IDC). Panels **(A, B)** show 15RS and 7.5RS treatment under SD1, respectively. Panels **(C, D)** show 15RS and 7.5RS treatment under SD2, respectively. Panels **(E, F)** show 15RS and 7.5RS treatment under SD3, respectively.

After linear regression of 14 factors (*X*_i_) with a substantial impact on the GY following each treatment, seven independent variables (action factors), including *X*_1_ (TP), *X*_2_ (TS), *X*_7_ (effective spikes on the main stem), *X*_9_ (percentage of effective tillers in spring), *X*_12_ (ESW), *X*_13_ (KN), and *X*_14_ (GW), were included in the regression model, and the DDC and IDC of X_i_ of the GY were determined (**Figures 12A–F**).

For SD1, four factors were included in the regression model of 15RS: *X*_1_ and *X*_9_ negatively affected GY (-1.011 in total), and *X*_7_ and *X*_13_ had a positive effect (0.931 in total). *X*_7_ had the most considerable indirect effect on GY through *X*_9_ (X_7 → 9_) (IDC = 0.465), followed by *X*_1 → 13_ and *X*_1 → 9_ (IDC mean 0.380). In the 7.5 RS treatment, *X*_13_, *X*_14_, *X*_12_, and *X*_1_ were the main factors affecting *GY* (all positive). The effects of X_13_ and X_14_ were significant (mean DDC = 1.097), and *X*_13_ was significantly related to GY (*P*<0.05). *X*_13_ and *X*_14_ strongly negatively affected GY (mean IDC = -0.852). *X*_13_ and *X*_1_ had significant positive and negative effects on GY through *X*_1_ (IDC = 0.646 and −0.655, respectively).

For SD2, *X*_13_ and *X*_14_ were included in the two-row spacing treatment return models. Compared with 15RS, the DDC value of *X*_13_ versus GY under 7.5RS treatment increased by 21.6%, whereas *X*_14_ versus *GY* decreased significantly. For 15RS and 7.5RS, *X*_13 → 2_ and *X*_13 → 9_ had the most substantial effects on GY (IDC = 0.393 and 0.465, respectively).

For SD3, seven independent variables (*X*_12_, *X*_13_, *X*_14_, *X*_1_, *X*_7_, *X_9_*, and *X*_14_) were included in 15RS and 7.5RS regression models. Among these, *X*_12_ and *X*_14_ in the 15RS and 7.5RS groups had the greatest effect on GY (DDC = 0.835 and −0.838, respectively). For the 15RS group, *X*_13 → 9_ had the strongest effect on GY (IDC = 0.498), whereas X_14 → 7_ of the 7.5RS group had the largest effect on GY (IDC = −0.269). Overall, the independent variable with the highest frequency of 15RS treatment in the regression model was *X*_13_, whereas those in the 7.5RS treatment were *X*_1_ and *X*_14_. The direct effect of *X*_13_ on GY increased with a delay in the sowing date, whereas that of *X*_14_ decreased. Under 15RS treatment, *X*_1_, *X*_13_, and *X*_7_ and *X*_1_, *X*_13_, and *X*_13_ in the SD1, SD2, and SD3 groups had the strongest direct effects on GY, whereas those for 7.5RS treatment were *X*_14_, *X*_13_, *X*_14_, *X*_13_, *X*_13_, and *X*_14_, respectively.

## 4 Discussion

### 4.1 Effect of optimized plant spatial competition on wheat tillering

Plants primarily compete for resources such as light, water, and nutrients (Sial et al., 2010). To fully use resources, plants must generate appropriate plant and leaf types to adapt to competition for living space (Evers et al., 2006; Dreccer et al., 2012; Fioreze and Rodrigues, 2014; Wang et al., 2018). However, as an essential aspect of the participation of gramineous crops in resource competition, the number of tillers and the start and end times of their formation are significantly affected by the distance between adjacent plants (Liu et al., 2011; Alzueta et al., 2012; Clerget et al., 2016; Lecarpentier et al., 2019). If the distribution of plants is not sufficiently uniform, it will lead to competition in the utilization of soil nutrients in areas with dense plant distribution, which will further lead to excessive growth. Further optimization of the allocation of plant row spacing to optimize crop spatial competition under conditions of equal density has attracted increasing research attention (Ballaré, 1999; Golubski et al., 2008; Liu et al., 2021c; Xiao et al., 2022).

Alzueta et al. (2012) reported that changing row spacing affects interactions between neighboring plants; such interactions involve competition for resources and active morphogenetic responses triggered by neighbors’ perceptions. De Vita et al. (2017) and Fischer et al. (2019) reported that changing the density by increasing row spacing has a stronger impact than simply changing the plant density in rows because it leads to increased spatial heterogeneity of crops. In another study, plant spacing was reduced under wide row spacing, and the shading reaction of wheat plants cultivated at high density appeared at the early tillering stage. This study also suggested that wheat plants respond to changes in the distance between rows by altering their tillers, leaf size, and angle (de Wit et al., 2012).

Row spacing and density currently used in wheat cultivation do not lead to competition between plant rows. For example, at a conventional sowing date, density, and row spacing in the NCP, the canopy of winter wheat is not closed in the pre-winter stage, and thus there is almost no competition between rows in the pre-winter stage. However, competition among plants exists objectively. In the tillering stage, wheat leaves contact each other successively, leading to spatial competition in signal response plants and further phenotypic changes. For example, Hussain et al. (2012) reported that decreasing wheat row spacing from 30 to 15 cm increased the tiller number by 79.3%. Abichou et al. also reported that wheat row spacing was reduced from 35 cm to 17.5 cm without changing the density, and the plant spacing was correspondingly expanded, increasing the number of tillers of different varieties by 12%–19%. Other studies have shown similar trends for rice (Clerget et al., 2016) and *Aegilops tauschii* Coss (Yu et al., 2020).

We reduced the row spacing of winter wheat in the NCP area from 15 cm to 7.5 cm. The tillering and biomass of single plants before winter increased by 14.5% and 19.1%, respectively, demonstrating the feasibility of reducing row spacing and expanding plant spacing to regulate tillering in wheat production. Moreover, after postponing the sowing date, reducing row spacing promoted an increase in the number of tillers and wheat biomass. This result is applicable to tillering control in late-sown winter wheat by controlling various factors. The detailed mechanism of the above phenomena was revealed from the perspective of phytohormones (see below). Carbohydrates, plant temperature, and radiation energy utilization will be reported in subsequent papers.

### 4.2 Effects of optimized plant spatial competition on phytohormones content in wheat

A variety of phytohormones are single or synergistic and play important roles in the growth and development of crop branches and tillers (Müller and Leyser, 2011; Cai et al., 2018; Wang et al., 2018). Auxin, GA, and IAA inhibit plant tillering to different degrees, whereas CTK and endogenous ZT promote plant tillering. Additionally, the growth of wheat tiller buds is regulated by the concentrations of IAA and ZT in the tillering node and the ratios of IAA to ZT and abscisic acid to ZT (Shimizu-Sato et al., 2008; Cai et al., 2018). SLs regulate the tillering of gramineous crops, possibly due to the interaction between SLs and auxin (Liang et al., 2010). SLs and auxin may regulate tillering, as auxin regulates branching by inducing SL synthesis, or SLs may systematically regulate auxin transport (Tanaka, 2006; Prusinkiewicz et al., 2009).

The factors influencing phytohormones and environmental conditions during plant tillering are complex. The effects of phytohormone type and content, plant sowing density, plant spacing configuration, and other factors on tillering have been widely examined (Evers et al., 2006; Müller and Leyser, 2011; Hussain et al., 2012; Wang et al., 2018; Kaplan and Specht, 2022). However, there is a lack of direct evidence for the role of hormones and whether competition between plants directly affects hormones. For example, hormones are internal factors that link plant density to tillering development in *A. tauschii* (a gramineous crop), but the specific roles of these hormones remain unknown. We showed that the IAA and GA contents of 7.5RS for the three sowing-date groups decreased significantly, whereas the ZR and SL contents increased significantly compared to those of 15RS. Previous research on gramineous crops, such as *A. tauschii* Coss (Yu et al., 2020) and *Miscanthus × giganteus* (Schmidt et al., 2017), showed that when the density increased, the IAA and GA contents increased, whereas the content of CTK, a growth hormone, showed the opposite trend. Although the above studies and studies on crops differ from the present study, the results support those of our study.

Multiple hormones affect competition for space between plants, including plant spacing and density, and regulate tiller growth (Yu et al., 2020). For example, GA inhibits tillering in tall fescue through crosstalk with CTK (Zhuang et al., 2019). Another study showed that plant hormone crosstalk, in which SL downregulated CTK levels by inducing the expression of CYTOKININ OXIDASE/DEHYDROGENASE 9, was involved in tillering regulation in rice (Duan et al., 2019). Additionally, natural auxin IAA stimulates SL biosynthesis, thus providing a potential mechanism for inhibiting bud outgrowth (Rameau et al., 2015).

We found that the number of tillers and biomass were significantly and positively correlated with SLs/GA for the three sowing-date groups (*P*<0.05), indicating that the environmental conditions changed when row spacing was reduced, which may inhibit the top development of wheat, promote the proportion of branching development hormones, and increase tillering. This study improves our understanding of the influence of plant spacing on endogenous hormones and their regulatory effects on tillering. However, we only evaluated the tillering node, and further in-depth analyses of the hormone characteristics of the root, other parts of the stem, and leaves are required. In addition, some hormones may not directly act on tillering but act as primary messengers, regulating tillering by changing the content of other hormones or the balance of several phytohormones (Cline and Oh, 2006), which requires further evaluation.

### 4.3 Effect of optimizing plant spatial competition on grain yield factors of wheat

Appropriate row spacing is important for improving crop productivity because plants growing in too wide rows may not efficiently utilize light, water, and nutrient resources, whereas growth in narrow rows may result in inter-row solid competition (Ali et al., 1999). Therefore, adjusting the plant and row spacing of crops and alleviating competition for plant growth space is important for cultivating crops with high yields, particularly closely planted crops with tillering characteristics (Abichou et al., 2019; Fischer et al., 2019). The yield of wheat with reduced row spacing (15 and 5 cm) was significantly higher than that at 25 cm (De Vita et al., 2017). Hussain et al. reported that wheat yield increased when row spacing was reduced, but there were large differences among varieties. Compared with the 30 cm and 25 cm row spacings, the yield of medium-plant height varieties was the highest at 20 cm, and that of short-plant height varieties was the highest at 15 cm, showing that the GW of wheat decreased after row spacing was reduced. This result corresponds to an increased number of spikes or grains per spike, thus increasing grain yield (Shimizu-Sato et al., 2008; Hussain et al., 2012).

Some studies have also pointed out that there is no evidence that increasing planting density can offset the broader row yield reduction (Fischer et al., 2019). Additionally, many studies have shown that reducing row spacing improves yield. However, when row spacing is reduced to a certain distance, the distribution of plant populations and individuals reaches a balance. If row spacing is further decreased, yield is not affected or even decreased (Liu et al., 2016; De Vita et al., 2017). However, the mechanism by which reducing row spacing can increase yield is complex. Decreasing row spacing can reduce the rectangularity of seed distribution (as the ratio of inter-row to intra-row distance), enabling greater uptake of soil nutrients and increasing ground cover by the crop (De Vita et al., 2017). Additionally, changing the density by increasing the inter-row distance has a stronger effect than changing the density of plants within a row, as it leads to increased crop spatial heterogeneity (Fischer et al., 2019). Furthermore, plants with a uniform spatial distribution show better tolerance to high-density planting, and the population and individuals receive more coordinated light, which is an important factor in reducing row spacing to improve yield (Liu et al., 2016).

We showed that reducing row spacing could significantly increase the number of pre-winters and total tillers. Row spacing, Y × SD interactions, and other sources of variation also had significant effects. The average yield of the three sowing-date groups increased by 6.3% when the row spacing was decreased from 15 cm to 7.5 cm (average of two years). The yield increase of the LSRE treatment was mainly reflected in the proportional increase of three yield factors, particularly the interaction between the grain number per spike and GW, which is different from the results of previous studies. According to path analysis (**Figure 12**), the number of grains per spike in 15RS had the most significant influence on yield, whereas 7.5RS had the greatest impact on GW. The contribution of GW to the 7.5RS yield increase increased when the sowing date was delayed. In addition, the pre-winter tillers of the three sowing-date groups in the 7.5RS treatment were included in the regression model, with the results supporting the importance of tillering in the LSRE treatment.

The tiller number was closely related to the changes in the contents of various phytohormone contents under LSRE treatments, specifically lower IAA and GA contents, higher ZR and SL contents, and higher SLs/GA (**Figure 11**). Although the phytohormone content in the pre-winter stage cannot directly control grain yield, the change in phytohormones significantly affects tillers, spikes the number of wheat, and further controls yield. Therefore, it is very necessary to study the change in hormone content as an important physiological mechanism of LSRE treatment to control wheat grain yield. In particular, the change in phytohormone content in the middle of the tillering stage of pre-winter has a more significant effect on the regulation of wheat tillering. It is necessary to continue studying.

## 5 Conclusion

Compared with the 15RS sowing date treatment, 7.5RS alleviated plant space competition, inhibited the accumulation of IAA and GA at the tillering nodes of wheat, increased the content of ZR and SLs, changed the IAA/ZR and SLs/GA, promoted tillering before winter, increased biomass accumulation, and delayed the sowing date more significantly. The increase in tillering and GW before winter was the main factor leading to increased yield in the LSER treatment. LSER can alter the phytohormone content, promote tiller growth, and increase the GW and grain yield of wheat in the NCP, particularly in the heat resource shortage area in the northern part of the NCP.

## Data availability statement

The original contributions presented in the study are included in the article/supplementary material. Further inquiries can be directed to the corresponding authors.

## Author contributions

WZ and MY conceived the project and set scientific objectives. BY, JG, LG and PL contributed to the preparation of the field experiment and data acquisition. BY, XL, and PL wrote the manuscript. All authors contributed to the article and approved the submitted version.
